# Supplementary material for: Oncologic Drugs Approval in Europe for Solid Tumors: Overview of the Last 6 Years
Source: Cancers (Basel). 2022 Feb 11;14(4):889. doi: 10.3390/cancers14040889 (PMC8870299; doi:10.3390/cancers14040889)
Supplement: Supplementary file 1 [file cancers-14-00889-s001.zip › cancers-1589830-supplementary.pdf]

cancers

MDPI

Table S1. Number of indications for single medicine.

DRUG	INDICATIONS (n)
pembrolizumab	13
nivolumab	10
atezolizumab	8
olaparib	6
ramucirumab	4
dabrafenib	4
cabozantinib	3
ribociclib	3
selpercatinib	3
avelumab	3
ipilimumab	3
bevacizumab	3
lenvatinib	3
fulvestrant	2
alectinib	2
osimertinib	2
ceritinib	2
rucaparib	2
crizotinib	2
abemaciclib	2
apalutamide	2
palbociclib	2
docetaxel	2
brigatinib	2
durvalumab	2
trastuzumab/pertuzumab sc	2
entrectinib	2
pertuzumab	2
niraparib	2
trifluridine/tipiracil	1
alpelisib	1
binimetinib	1
Irinotecan (pegylated liposomal formulation)	1
trametinib	1
larotrectinib	1
abiraterone acetato	1
dacomitinib	1
eribulin	1
lorlatinib	1
talazoparib	1
lutetium (177lu) oxodotreotide	1
trastuzumab emtansine	1
nab-paclitaxel	1
tucatinib	1
necitumumab	1
neratinib	1
regorafenib	1
cemiplimab	1
everolimus	1
avapritinib	1
sonidegib	1
afatinib	1
tivozanib	1
olaratumab	1
trastuzumab deruxtecan	1
encorafenib	1
cobimetinib	1
paclitaxel	1
trifluridine / tipiracil	1
darolutamide	1
vandetanib	1

panitumumab	1
enzalutamide	1
**Total**	**132**

Table S2. PFS and OS Hazard ratio for disease/setting and type of drug.

Disease	Angiogenesis Inhibitor	Chemotherapy	Hormone	ICI	Signal transduction inhibitor	Total
**cervix carcinoma**	**0.74**					**0.74**
advanced	0.74					0.74
**breast cancer**				**0.71**		**0.71**
advanced				0.71		0.71
**CRC**	**0.84**	**0.68**		**0.77**	**0.52**	**0.70**
advanced	0.84	0.68		0.77	0.52	0.70
**gastric cancer**		**0.69**				**0.69**
advanced		0.69				0.69
**HCC**	**0.76**			**0.58**		**0.72**
advanced	0.76			0.58		0.72
**HNSCC**				**0.66**		**0.66**
advanced				0.66		0.66
**liposarcoma**		**0.77**				**0.77**
advanced		0.77				0.77
**melanoma**				**0.63**	**0.71**	**0.65**
advanced				0.63	0.71	0.65
**NSCLC**	**0.86**			**0.67**	**0.84**	**0.70**
non advanced				0.52		0.52
advanced	0.86			0.68	0.84	0.72
**oesophageal squamous cell carcinoma**				**0.77**		**0.77**
advanced				0.77		0.77
**ovarian cancer**	**0.82**					**0.82**
advanced	0.82					0.82
**pancreatic cancer**		**0.67**				**0.67**
advanced		0.67				0.67
**prostate cancer**		**0.81**	**0.65**			**0.70**
advanced		0.81	0.65			0.70
**RCC**				**0.68**		**0.68**
advanced				0.68		0.68
**SCLC**				**0.76**		**0.76**
advanced				0.76		0.76
**urothelial carcinoma**				**0.76**		**0.76**
advanced				0.76		0.76
**Total**	**0.79**	**0.72**	**0.65**	**0.68**	**0.69**	**0.70**
